# The association between dietary inflammation index and bone mineral density: results from the United States National Health and nutrition examination surveys

**DOI:** 10.1080/0886022X.2023.2209200

**Published:** 2023-05-08

**Authors:** Siyao Li, Mengru Zeng

**Affiliations:** Department of Nephrology, Hunan Key Laboratory of Kidney Disease and Blood Purification, The Second Xiangya Hospital, Central South University, Changsha, China

**Keywords:** Dietary inflammatory index, BMD, osteoporosis, femur bone

## Abstract

**Objective:**

To investigate the associations of dietary inflammation index (DII) with bone density and osteoporosis in different femoral areas.

**Methods:**

The study population was selected from the National Health and Nutrition Examination Survey (NHANES) with the exclusion criteria of age 18, pregnancy, or missing information on DII, femoral bone marrow density (BMD), estimated glomerular filtration rate (eGFR), and urine albumin-to-creatinine ratio (UACR), or had diseases which may influence systemic inflammation. DII was calculated based on the questionnaire interview of dietary recall within 24 h. Subjects’ baseline characteristics were collected. The associations between DII and different femoral areas were analyzed.

**Results:**

After applying exclusion criteria, 10,312 participants were included in the study. Significant differences among DII tertiles were found in BMD or T scores (*p* < .001) of the femoral neck, the trochanter, the intertrochanter, and the total femur. High DII was associated with low BMDs and T scores in all the femoral areas (all *p* < .01). Compared to low DII (tertile1, DII < 0.380 as reference), in the femoral neck, the intertrochanter, and the total femur, increased DII is independently associated with increased the possibility of the presence of osteoporosis (OR, 95% CI: 1.88, 1.11–3.20; 2.10, 1.05–4.20; 1.94, 1.02–3.69, respectively). However, this positive association was only observed in the trochanteric area of the non-Hispanic White population after full adjustment (OR, 95% CI: 3.22 (1.18, 8.79)). No significant difference in the association of DII and the presence of osteoporosis were found in subjects with or without impaired kidney function (eGFR < 60 ml/min/1.73 m^2^).

**Conclusion:**

High DII is independently related to declined femoral BMD of femoral areas.

## Introduction

Osteoporosis, with the characteristics of declined bone mass and degraded bone microstructure, is considered a common senile disease that could lead to an increased risk of bone fragility and fracture, and contribute to an increased mortality in patients [1]. With an estimated 200 million people affected, osteoporosis has been a major global public health concern [2]. As an essential determinant of bone health, the measurement of bone mineral density (BMD) is a common operational tool for the diagnosis of osteoporosis [3].

Chronic systemic inflammation was found not only to contribute to an elevated risk of osteoporosis and fragility fractures but also has a close correlation with some of the critical factors in bone physiology [4–6]. Studies have shown that elevated inflammatory markers in circulation may predict bone loss and resorption in the elderly [7]. Others have demonstrated that higher serum inflammatory markers may be a potential risk factor for fractures in older women and men [5]. These results all support the conclusion that between inflammation and osteoporosis, there should be a close relationship.

Currently, in addition to pharmacological approaches, non-pharmacological approaches such as suitable diet patterns, and adopting a physically active lifestyle, are also major inventions that may help with osteoporosis treatments and prevention [8]. Nutrients from daily diets, such as unsaturated fatty acids, vitamins, fibers, and proteins, can regulate bone metabolism and help with bone health [8–10].

However, dietary components have been considered to have both pro-inflammatory and anti-inflammatory potential which may influence systemic inflammation [11–13]. Growing evidence has shown that dietary patterns are closely associated with BMD and fracture risk in older adults, especially in women [14–16]. Therefore, a proper diet with appropriate inflammatory potential is essential for maintaining bone mass.

The dietary inflammatory index (DII), a scoring system derived from ∼ 2000 literature and based on 11 food consumption data sets worldwide, is considered a good strategy for the qualification of a diet’s inflammatory potential [17]. A positive DII value indicates pro-inflammatory potential while a negative value represents anti-inflammatory potential. The higher the absolute value of DII, the stronger the effect of diet on inflammation. DII has been reported to strongly correlate with many chronic diseases, such as coronary heart disease [18], metabolic syndrome [19], cancer [20], and osteoarthritis [21]. Studies demonstrated that a low DII diet was related to improved BMD in postmenopausal women of different races [22–24]. A meta-analysis based on a large population from multiple studies also indicated that a diet pattern with high pro-inflammatory potential significantly correlates with decreased lumbar and hip BMD and is a risk factor of osteoporosis and fractures in these areas [25]. However, such an association between higher DII and worse BMD was rarely found in men [24,26,27], only one study showed that a pro-inflammatory diet was linked with an increased possibility of hip osteoporosis and fracture in both Chinese men and women [28]. Nevertheless, the associations between DII and osteoporosis in require further exploration.

Among the fractures caused by osteoporosis, hip fracture is the most severe one [29], which is more often observed and results in more severe consequences in patients [30,31]. For the present study, the hip area was chosen for BMD measurement. Using a large multiracial cohort from the National Health and Nutrition Examination Survey (NHANES), we conducted this cross-sectional study to explore the association between the DII and the BMD of the femoral neck, the trochanter, the intertrochanter, and the total femur, as well as the possibility of diagnosed osteoporosis in these areas, which may bring future benefits to prevention and treatment of osteoporosis.

## Materials and methods

### Study design, setting, and subjects

The NHANES program is a series of continuous national surveys conducted by the National Center for Health Statistics (NCHS, Hyattsville, MD, USA), which selected a nationally representative sample to examine the nutritional and health conditions of the non-institutionalized civilian population of the United States using a complex, stratified, multistage probability sampling design [32]. The NCHS authorized the survey procedure, and all subjects provided signed informed consent. NHANES provides detailed information over the website, https://www.cdc.gov/nchs/nhanes/index.htm.

**Figure 1.**
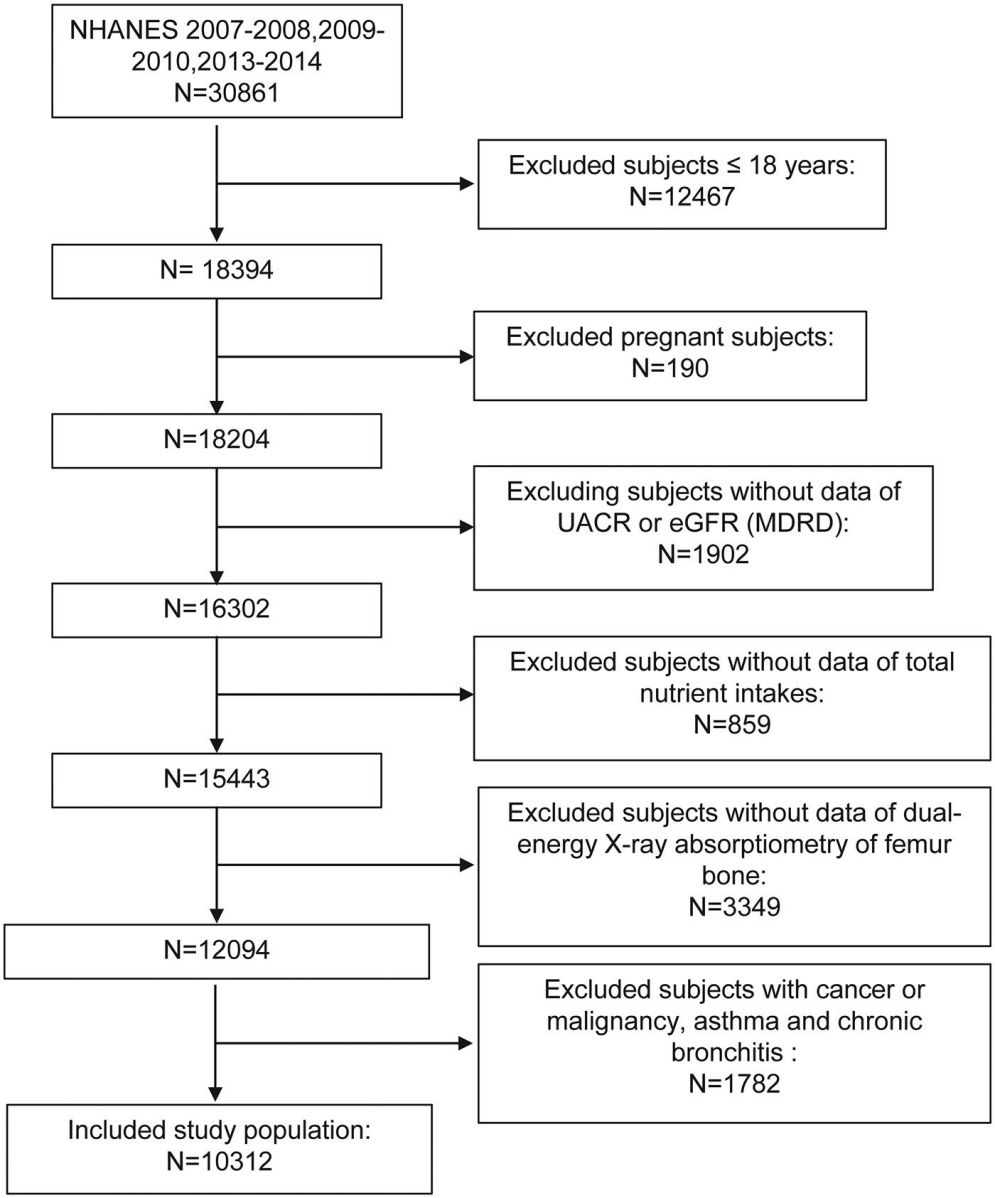
Study population.

To investigate the relationship between DII and the femoral BMD and the presence of osteoporosis, we performed this study. Measurement conduction and data record were all finished by staff from the NHANES. All the data were collected from the NHANES 2007–2010 and 2013–2014, in total 3 cycles. As shown in Figure 1, participants who are younger than or equal to18 years old, pregnant at the screen, or had incomplete data of total nutrition intakes, dual-energy X-ray absorptiometry of femur bone, estimated glomerular filtration rate (eGFR), and urine albumin-to-creatinine ratio (UACR), or had cancer or malignancy, asthma, and chronic bronchitis which may influence systemic inflammation were excluded. after applying exclusion criteria, 10,312 participants were included in the study.

### Calculation of DII

DII score was calculated using the daily dietary information of every participant and was the exposure variable in this study. Based on robust literature, the complete DII score system contains 45 different food parameters that have pro- or anti-inflammatory properties to calculate the overall influence of a diet on systemic inflammation [17]. Though only 27 or 28 of the 45 food parameters were recorded in the NHANES database, previous studies confirmed that DII scores based on <30 nutrients are still reliable [17,33,34]. The parameters used for DII calculation in this study included energy, carbohydrate; protein; total fat; dietary fiber; cholesterol; saturated, monounsaturated, and polyunsaturated fatty acids; ω-3 and ω-6 polyunsaturated fatty acids; vitamin A, B1, B2, B3, B6, B12, C, D, and E; folic acid; alcohol; β-carotene; caffeine; iron; magnesium; zinc; and selenium. A positive or negative DII value represents a pro-inflammatory or anti-inflammatory diet, respectively.

### BMD measurement and osteoporosis diagnosis

BMD was measured by dual-energy X-ray absorptiometry (DXA), data of the femoral neck, the trochanter, the intertrochanter, and the total femur were collected. The detailed DXA measurement agreement is publicly available at http://www.cdc.gov/nchs/nhanes/. Under the guidance of the |World Health Organization criteria, all the BMDs were converted into T-scores using the formula: T-score = (BMD respondent - mean BMD reference group)/SD reference group [35,36]. Osteopenia is diagnosed as a subject whose T score is less than −1.0 but more than −2.5, while osteoporosis is diagnosed as a subject whose T score is less than or equal to −2.5 [27,37].

### Covariate ascertainment

Categorical variables were stated as follows: gender (male, female); race/ethnicity (Mexican American, non-Hispanic White, non-Hispanic Black, other race/ethnicity); Education (less than high school, high school diploma, more than high school); Income was evaluated by the poverty income ratio (PIR, PIR <1, poor; PIR 1–3, near-poor; PIR ≥3, not poor) [38]. Smokers were defined as subjects who reported smoking more than 100 cigarettes during their lifetime [39]; Cardiovascular disease (CVD) was defined as “the doctor told you had congestive heart failure, coronary heart disease, angina/angina pectoris, heart attack, or a stroke”; hypertension was defined as systolic blood pressure ≥ 140 mm Hg or diastolic blood pressure ≥ 90 mm Hg or now taking prescribed medicine for high blood pressure. Diabetes mellitus (DM) was defined as “the doctor told you had diabetes” or taking diabetic pills to lower blood sugar now or taking insulin now or fasting plasma glucose ≥ 126 mg/dL or 75-g oral glucose tolerance test of at least 200 mg/dL or glycohemoglobin ≥ 6.5%; Liver condition was defined as “the doctor told you had a liver condition”; Hypercholesterolemia was defined as “the doctor told you had hypercholesterolemia”; Arthritis was defined as “a doctor told you had arthritis”. The use of medication was categorized as “yes” or “no”.

Continuous variables were stated as follows: Age was an age in years at screening. Dietary calcium intake was collected from the dietary intake information by 24 h dietary recalls. Blood pressure data were defined as the average values of all available systolic blood pressure (SBP) and diastolic blood pressure (DBP) values collected when the subjects visit the examination center. The measurements of white blood cell (WBC) count, neutrophil count, lymphocyte count, serum albumin, serum fast hemoglobin, serum glycohemoglobin, serum calcium, serum phosphorus, and serum c-reactive protein were used. Body mass index (BMI) information was collected from the body measurement data at the screen; For the estimated glomerular filtration rate (eGFR) calculation, serum creatinine across different time periods was calibrated as the NHANES recommended [40] and the Modification of Diet in Renal Disease Study Equation [41]were used. Relevant laboratory methods and detailed descriptions can be found on this website, http://www.cdc.gov/nchs/nhanes/.

It is noteworthy that dietary alcohol and vitamin D intake were included in the DII score and were not used as covariates again.

### Statistical analysis

Statistical analysis was performed under the guidance of the Centers for Disease Control and Prevention (CDC) (https://wwwn.cdc.gov/nchs/nhanes/tutorials/default.aspx). *p* < .05 was considered significant. 95% confidence intervals (CI) were also shown.

To describe the baseline characteristics of subjects by tertiles of DII (< 0.380, 0.380 − 2.286, and > 2.286). Normally distributed continuous variables were represented as means ± standard deviation (SD) and the differences among tertiles were calculated by ANOVA, non-normally distributed continuous variables were represented as Median (interquartile range), and the differences among tertiles were calculated by Kruskal–Wallis *H* Test, while categorical variables were represented as a number with percentages and the differences among tertiles were calculated by the chi-square test. Fisher’s exact test was introduced to compute the significance of the difference if the count variable has a theoretical value of < 10.

Since there may be some possible confounding factors, the weighted linear regression models with adjustments were introduced to compare the BMD levels, T score levels, and osteoporosis incidence among different tertiles of DII. Model 1 was adjusted for age, gender, race/ethnicity; Model 2 was adjusted for age, gender, race/ethnicity, smoker, BMI, eGFR, UACR, serum C-reactive protein, WBC count, NLR, serum calcium, arthritis, aspirin use, calcitonin use, biphosphonate use, DMARDs use, calcium intake and estrogen use. Data presented as *β* or odds ratio (OR) with 95% CI. Subgroup analyses were also conducted.

All analyses and graphic programs were performed with the statistical software package R-3.4.3 (https://www.R-project.org, The R Foundation) and Empower-Stats (https://www.empowerstats.com, X&Y Solutions, Inc., Boston, MA).

## Results

### Baseline characteristics

A total of 10,312 subjects were included in this study after the exclusion of those with age ≤18 years old, pregnant, or loss information of eGFR, UACR, nutrient intake or bone density, or disease that may influence systemic inflammation including cancer or malignancy, asthma, and chronic bronchitis. after (Figure 1). The baseline characteristics of the whole study population and subjects by tertiles of DII are given in Table 1. The medium age of the population is 50.0 and 50.81% were men. Most of the subjects were obese (median (interquartile range) BMI: 27.59 (24.15–31.47) kg/m^2^]. Regarding the metabolic syndrome, though the blood pressure was not high (120.67 mm Hg of medium SBP, and 69.33 mm Hg of medium DBP when measured at participation), nearly half of the subjects has a hypertension history (40.14%). What’s more, over half of the population has DM (58.57%) or hypercholesterolemia (58.68%), while CVD (7.79%), and liver condition (3.10%) were not very common.

**Table 1.** Baseline characteristics of the study population by tertiles of dietary inflammatory index.

		Dietary Inflammatory Index (DII)			
	total	Tertile1 < 0.380	Tertile2 0.380–2.286	Tertile3 > 2.286	*p*-value
N	10312	3437	3437	3438	
Age (years)	50.00 (37.00–63.00)	50.00 (38.00–62.00)	50.00 (37.00–63.00)	50.00 (36.00–65.00)	0.22
Gender, *n*(%)					<.001*
male	5343 (51.81%)	2168 (63.08%)	1783 (51.88%)	1392 (40.49%)	
female	4969 (48.19%)	1269 (36.92%)	1654 (48.12%)	2046 (59.51%)	
Race/ethnicity, *n*(%)					<.001*
Mexican American	1957 (18.98%)	687 (19.99%)	661 (19.23%)	609 (17.71%)	
Non-Hispanic White	1858 (18.02%)	612 (17.81%)	635 (18.48%)	611 (17.77%)	
Non-Hispanic Black	4623 (44.83%)	1645 (47.86%)	1522 (44.28%)	1456 (42.35%)	
Other race	1874 (18.17%)	493 (14.34%)	619 (18.01%)	762 (22.16%)	
Education level, more than high school, *n*(%)	5092 (49.44%)	1966 (57.22%)	1723 (50.19%)	1403 (40.90%)	.001*
Income, *n*(%)					<.001*
Poor	1887 (20.00%)	520 (16.56%)	581 (18.47%)	786 (24.94%)	
Near poor	3914 (41.48%)	1171 (37.29%)	1327 (42.19%)	1416 (44.92%)	
Not poor	3636 (38.53%)	1449 (46.15%)	1237 (39.33%)	950 (30.14%)	
Smoker, *n*(%)	4575 (45.32%)	1458 (43.33%)	1526 (45.31%)	1591 (47.34%)	.004*
BMI, (kg/m^2^)	28.25 ± 5.69	27.85 ± 5.42	28.32 ± 5.72	28.56 ± 5.91	<.001*
Systolic blood pressure (mm Hg)	123.55 ± 18.22	122.82 ± 17.34	123.22 ± 17.57	124.60 ± 19.62	<.001*
Diastolic blood pressure(mm Hg)	68.18 ± 13.91	69.06 ± 13.73	68.21 ± 13.41	67.26 ± 14.51	<.001*
Femoral neck BMD	0.83 ± 0.15	0.84 ± 0.15	0.83 ± 0.15	0.82 ± 0.16	<.001*
Femoral neck T score	−0.30 (−1.20 to 0.60)	−0.20 (−1.10 to 0.60)	−0.30 (−1.10 to 0.60)	−0.40 (−1.20 to 0.50)	<.001*^,^**
Trochanter BMD	0.73 ± 0.13	0.75 ± 0.13	0.73 ± 0.13	0.71 ± 0.13	<.001*
Trochanter T score	0.20 (−0.70 to 1.10)	0.30 (−0.60 to 1.30)	0.20 (−0.70 to 1.10)	0.00 (−0.90 to 0.90)	<.001*^,^**
Intertrochanter BMD	1.15 ± 0.19	1.17 ± 0.18	1.16 ± 0.19	1.13 ± 0.19	<.001*
Intertrochanter T score	0.40 (−0.50 to 1.30)	0.60 (−0.30 to 1.50)	0.50 (−0.40 to 1.30)	0.30 (−0.60 to 1.20)	<.001*^,^**
Total femur BMD	0.97 ± 0.16	0.99 ± 0.16	0.98 ± 0.16	0.95 ± 0.16	<.001*
Total femur T score	0.20 (−0.60 to 1.10)	0.40 (−0.50 to 1.30)	0.30 (−0.60 to 1.20)	0.10 (−0.80 to 1.00)	<.001*^,^**
eGFR(ml/min/1.73 m^2^)	93.27 (78.38–109.78)	92.66 (78.75–109.04)	93.49 (78.78–109.47)	93.44 (77.53–111.00)	.50
UACR(mg/g)	0.69 (0.44–1.29)	0.62 (0.42–1.15)	0.69 (0.44–1.25)	0.76 (0.48–1.53)	<.001*,**
Serum albumin(g/dL)	42.62 ± 3.14	43.04 ± 3.13	42.67 ± 3.04	42.15 ± 3.18	<.001*
Fast serum glucose (mg/dL)	93.00 (85.00–104.00)	93.00 (86.00–104.00)	93.00 (85.00–104.00)	93.00 (85.00–105.000	.24
Glycohemoglobin (%)	5.50 (5.20–5.90)	5.50 (5.20–5.80)	5.50 (5.20–5.90)	5.50 (5.30–5.90)	<.001*
Serum calcium (mmol/L)	2.35 (2.30–2.42)	2.35 (2.30–2.42)	2.35 (2.30–2.42)	2.35 (2.30–2.42)	.003*
Serum phosphorus (mmol/L)	1.20 (1.10–1.32)	1.20 (1.10–1.32)	1.23 (1.10–1.32)	1.20 (1.07–1.32)	.32
Serum C-reactive protein(mg/dL)	0.17 (0.07–0.39)	0.14 (0.06–0.31)	0.18 (0.07–0.39)	0.21 (0.08–0.48)	<.001*
WBC count (103 cells/uL).	6.80 (5.60–8.20)	6.65 (5.60–8.00)	6.90 (5.70–8.20)	6.90 (5.70–8.40)	<.001*
NLR	1.91 (1.45–2.53)	1.89 (1.45–2.50)	1.92 (1.44–2.50	1.92 (1.45–2.57)	.22
CVD, *n*(%)	591 (7.79%)	147 (6.07%)	197 (7.73%)	247 (9.43%)	<.001*
Hypertension, *n*(%)	4139 (40.14%)	1270 (36.95%)	1347 (39.19%)	1522 (44.27%)	<.001*
DM, *n*(%)	6040 (58.57%)	1983 (57.70%)	1976 (57.49%)	2081 (60.53%)	.017*
Liver condition, *n*(%)	235 (3.10%)	91 (3.76%)	73 (2.87%)	71 (2.71%)	0.07
Hypercholesterolemia, *n*(%)	5708 (58.68%)	1895 (58.63%)	1924 (59.42%)	1889 (58.00%)	0.51
Calcium intake (mg)	815.00 (525.75–1185.00)	1128.00 (820.00–1559.00)	818.00 (569.00–1138.00)	541.00 (350.00–803.00)	<.001*
Arthritis, *n*(%)	2570 (25.51%)	791 (23.55%)	828 (24.61%)	951 (28.36%)	<.001*
Aspirin use, *n*(%)	161 (2.88%)	38 (2.13%)	52 (2.79%)	71 (3.65%)	.021*
Calcitonin use, *n*(%)	6 (0.11%)	2 (0.11%)	2 (0.11%)	2 (0.10%)	1.00
Biphosphonate use, *n*(%)	180 (3.22%)	53 (2.97%)	63 (3.39%)	64 (3.29%)	.76
DMARDs use, *n*(%)	59 (1.06%)	12 (0.67%)	18 (0.97%)	29 (1.49%)	.046*
Estrogen use, *n*(%)	87 (1.04%)	24 (0.82%)	26 (0.94%)	37 (1.39%)	.09

Mean ± standard deviation (SD)and ANOVA are for continuous variables with a normal distribution. Median (interquartile range) and Kruskal–Wallis *H* Test are for continuous variables that are not normally distributed. N and percentage (%) and weighted *χ*^2^ test are for categorical variables.

DII: dietary inflammation index; BMI: body mass index; BMD: bone mineral density; eGFR: estimated glomerular filtration rate; UACR: urine albumin-creatinine ratio; WBC: white blood cell; NLR: neutrophil to lymphocyte ratio; CVD: cardiovascular disease; DM: diabetes mellitus; DMARDs, disease-modifying antirheumatic drugs.

*p < .05.

**p value was calculated by Fisher’s exact test.

People who had higher DII were more female, less educated, lower in income, higher in BMI, and smokers (all *p* < .01). They also had a higher prevalence of hypertension, DM, and arthritis, and a higher systolic and diastolic blood pressure, serum C-reactive protein, and WBC count, but has lower serum albumin and calcium intake (all *p* < .05). No significant difference in serum phosphorus was observed among different levels of DII. Across the tertiles of the DII, we found statistically significant associations with BMDs or T scores of all femoral regions (*p* < .001). For all the BMDs and T scores of all femoral areas, higher DII was associated with lower BMD and T scores (all *p* < .001).

#### Association of DII with femoral BMD and T score

We further analyzed the BMDs (Table 2) and T scores (Table 3) of different femur regions including the femoral neck, the trochanter, the intertrochanter, and the total femur based on the tertiles of DII by weighted linear regression models with different adjustment. A higher DII score was found to have a significant association with more BMD loss in all the studied femoral areas (all *p* < .0001) (Table 2). This association still exists after adjustment for age, gender, race/ethnicity, smoker, BMI, eGFR, UACR, serum C-reactive protein, WBC count, NLR, serum calcium, arthritis, aspirin use, calcitonin use, biphosphonate use, DMARDs use, calcium intake and estrogen use. Similarly, there was a positive correlation between higher DII and lower T scores in all the femoral areas (all *p* < .0001) (Table 3).

**Table 2.** The association betwwen DII and femoral BMD.

		Dietary Inflammatory Index (DII)				
			Tertile2 0.380–2.286		Tertile3 > 2.286	
Region of interest	Model	Tertile1 < 0.380	β (95% CI)	*p* value	β (95% CI)	*p* value
Femoral neck	Crude model	Reference	−0.01 (−0.01, 0.00)	.07	−0.02 (−0.03, −0.01)	<.0001*
	Adjusted model 1	Reference	−0.00 (−0.01, 0.00)	.36	−0.01 (−0.02, −0.00)	.0008*
	Adjusted model 2	Reference	−0.01 (−0.02, 0.00)	.15	−0.02 (−0.03, −0.01)	.0036*
Trochanter	Crude model	Reference	−0.02 (−0.02, −0.01)	<.0001*	−0.03 (−0.04, −0.03)	<.0001*
	Adjusted model 1	Reference	−0.01 (−0.01, −0.00)	.006*	−0.02 (−0.02, −0.01)	<.0001*
	Adjusted model 2	Reference	<0.0001*	.0281*	−0.02 (−0.03, −0.01)	.0004*
Intertrochanter	Crude model	Reference	−0.02 (−0.02, −0.01)	.0004*	−0.04 (−0.05, −0.03)	<.0001*
	Adjusted model 1	Reference	−0.00 (−0.01, 0.00)	.30	−0.02 (−0.02, −0.01)	<.0001*
	Adjusted model 2	Reference	−0.01 (−0.02, 0.00)	.16	−0.02 (−0.03, −0.01)	.0043*
Total femur	Crude model	Reference	−0.02 (−0.02, −0.01)	<.0001*	−0.04 (−0.05, −0.03)	<.0001*
	Adjusted model 1	Reference	−0.01 (−0.01, 0.00)	.11	−0.02 (−0.02, −0.01)	<.0001*
	Adjusted model 2	Reference	−0.01 (−0.02, 0.00)	.06	−0.02 (−0.03, −0.01)	.0005*

Adjusted model 1: Adjust for age, gender, race/ethnicity.

Adjusted model 2: Adjust for age, gender, race/ethnicity, smoker, BMI, eGFR, UACR, serum C-reactive protein, WBC count, NLR, serum calcium, arthritis, aspirin use, calcitonin use, biphosphonate use, DMARDs use, calcium intake and estrogen use.

BMD: bone mineral density; DII: dietary inflammation index; OR: odds ratio; CI: confidence interval; BMI: body mass index; eGFR: estimated glomerular filtration rate; UACR: urine albumin-creatinine ratio; WBC: white blood cell; NLR: neutrophil to lymphocyte ratio; DMARDs: disease-modifying antirheumatic drugs.

*p<.05.

**Table 3.** The association between DII and femoral T score.

		Dietary Inflammatory Index (DII)				
			Tertile2 0.380–2.286		Tertile3 > 2.286	
Region of interest	Model	Tertile1 < 0.380	*β* (95% CI)	*p* value	*β* (95% CI)	*p* value
Femoral neck	Non-adjusted	Reference	−0.06 (−0.12, 0.00)	.07	−0.18 (−0.24, −0.12)	<.0001*
	Adjust I	Reference	−0.02 (−0.07, 0.03)	.37	−0.09 (−0.14, −0.04)	.0008*
	Adjust II	Reference	−0.06 (−0.14, 0.02)	.15	−0.13 (−0.22, −0.04)	.0042*
Trochanter	Non-adjusted	Reference	−0.16 (−0.23, −0.10)	<.0001*	−0.34 (−0.41, −0.28)	<.0001*
	Adjust I	Reference	−0.08 (−0.14, −0.02)	.0065*	−0.17 (−0.23, −0.11)	<.0001*
	Adjust II	Reference	−0.11 (−0.20, −0.01)	.0312*	−0.19 (−0.29, −0.08)	.0004*
Intertrochanter	Non-adjusted	Reference	−0.11 (−0.17, −0.05)	.0004*	−0.30 (−0.36, −0.23)	<.0001*
	Adjust I	Reference	−0.03 (−0.08, 0.03)	.30	−0.11 (−0.17, −0.06)	<.0001*
	Adjust II	Reference	−0.06 (−0.15, 0.02)	.16	−0.14 (−0.24, −0.05)	.0038*
Total femur	Non-adjusted	Reference	−0.13 (−0.19, −0.06)	<.0001*	−0.31 (−0.38, −0.25)	<.0001*
	Adjust I	Reference	−0.04 (−0.10, 0.01)	.11	−0.13 (−0.19, −0.08)	<.0001*
	Adjust II	Reference	−0.08 (−0.17, 0.00)	.06	−0.17 (−0.26, −0.07)	.0004

Adjusted model 1: Adjust for age, gender, race/ethnicity.

Adjusted model 2: Adjust for age, gender, race/ethnicity, smoker, BMI, eGFR, UACR, serum C-reactive protein, WBC count, NLR, serum calcium, arthritis, aspirin use, calcitonin use, biphosphonate use, DMARDs use, calcium intake and estrogen use.

DII: dietary inflammation index; CI: confidence interval; BMI: body mass index; eGFR: estimated glomerular filtration rate; UACR: urine albumin-creatinine ratio; WBC: white blood cell; NLR: neutrophil to lymphocyte ratio; DMARDs: disease-modifying antirheumatic drugs.

*p<.05.

### Association of DII with femoral osteoporosis

We further investigated whether the DII score is associated with the possibility of the presence of osteoporosis in the femoral neck, the trochanteric, the intertrochanteric, and the total femoral areas (Table 4). Compared to low DII (tertile1, DII < 0.380 as reference), in the femoral neck, the intertrochanter, and the total femur, increased DII is independently associated with an increased possibility of the presence of osteoporosis (OR, 95% CI: 1.88,1.11–3.20; 2.10, 1.05–4.20; 1.94,1.02–3.69, respectively). However, this positive association was not observed in the trochanteric area exists after full adjustment (OR, 95% CI: 1.62, 0.85–3.09).

**Table 4.** The association between DII and the possibility of the presence of osteoporosis.

		Dietary Inflammatory Index (DII)				
			Tertile2 0.380–2.286		Tertile3 > 2.286	
Region of interest	Model	Tertile1 < 0.380	OR (95% CI)	*p* value	OR (95% CI)	*p* value
Femoral neck	Non-adjusted	Reference	1.20 (0.89, 1.62)	.22	1.81 (1.37, 2.39)	<.0001*
	Adjust I	Reference	1.06 (0.77, 1.45)	.72	1.31 (0.97, 1.75)	.0774
	Adjust II	Reference	1.34 (0.80, 2.26)	.27	1.88 (1.11, 3.20)	.019*
Trochanter	Non-adjusted	Reference	1.82 (1.17, 2.83)	.0078*	2.58 (1.70, 3.93)	<.0001*
	Adjust I	Reference	1.55 (0.98, 2.43)	.06	1.65 (1.07, 2.55)	.0222*
	Adjust II	Reference	1.49 (0.80, 2.81)	.21	1.62 (0.85, 3.09)	.1457
Intertrochanter	Non-adjusted	Reference	1.41 (0.86, 2.32)	.17	2.62 (1.68, 4.10)	<.0001*
	Adjust I	Reference	1.20 (0.72, 1.99)	.48	1.70 (1.07, 2.70)	.0245
	Adjust II	Reference	1.06 (0.52, 2.18)	.87	2.10 (1.05, 4.20)	.0365*
Total femur	Non-adjusted	Reference	1.22 (0.79, 1.89)	.37	2.30 (1.56, 3.40)	<.0001
	Adjust I	Reference	1.02 (0.65, 1.60)	.94	1.47 (0.98, 2.20)	.065
	Adjust II	Reference	1.20 (0.62, 2.30)	.59	1.94 (1.02, 3.69)	.0428*

Adjusted model 1: Adjust for age, gender, race/ethnicity.

Adjusted model 2: Adjust for age, gender, race/ethnicity, smoker, BMI, eGFR, UACR, serum C-reactive protein, WBC count, NLR, serum calcium, arthritis, aspirin use, calcitonin use, biphosphonate use, DMARDs use, calcium intake and estrogen use.

DII: dietary inflammation index; OR: odds ratio; CI: confidence interval; BMI: body mass index; eGFR: estimated glomerular filtration rate; UACR: urine albumin-creatinine ratio; WBC: white blood cell; NLR: neutrophil to lymphocyte ratio; DMARDs: disease-modifying antirheumatic drugs.

*p<.05.

Intriguingly, subgroup analysis (Table 5) demonstrated a positive correlation between higher DII and increased possibility of the presence of osteoporosis in the trochanteric area of the non-Hispanic White population, indicating that they may pay more attention to the BMD of this area than the other races (Table 5, Supplemental Figure 2). Other results of the subgroup analysis were consistent with the preliminary results. Though we have observed that the declined eGFR was related to a decreased BMD (Supplemental Figure 1) and increased chance of the presence of osteoporosis (Supplemental Table 2), no significant difference in the association between DII and the presence of osteoporosis was found when we compare the subjects with impaired kidney function (eGFR < 60 mL/min/1.73 m^2^) with those who have better kidney function (eGFR ≥ 60 mL/min/1.73 m^2^). We also made a subgroup analysis by vitamin D intake for the association between the DII without vitamin D and the presence of femoral osteoporosis (Supplemental Table 1), due to that vitamin D is closely related to bone health. No significant results were found in the trochanter, intertrochanter, and total femur, but an increased risk of the presence of osteoporosis in the femoral neck was found in subjects with high DII without vitamin D when vitamin D intake ≥3.2 µg/d.

**Table 5.** Subgroup analysis of the association between DII and the possibility of the presence of osteoporosis.

			Dietary Inflammatory Index (DII)					
Region of		Sample	Tertile1	Tertile2 0.380–2.286		Tertile3 > 2.286		*p* for
interest	Subgroup	size	< 0.380	OR (95% CI)	*p* value	OR (95% CI)	*p* value	interaction
Femoral neck	Age (year)							.248
	<65		Reference	0.62 (0.22, 1.73)	.3631	0.99 (0.37, 2.69)	.9854	
	≥65		Reference	1.65 (0.90, 3.03)	.1072	2.20 (1.18, 4.09)	.013*	
	Gender							.1458
	Male^a^	5343	Reference	1.34 (0.56, 3.21)	.5179	0.90 (0.33, 2.49)	.843	
	Female	4969	Reference	1.37 (0.71, 2.64)	.3452	2.31 (1.21, 4.42)	.0113*	
	Race/ethnicity							.9379
	Non-Hispanic White	4623	Reference	1.34 (0.68, 2.66)	.3965	2.01 (1.00, 4.03)	.0486*	
	Others	5689	Reference	1.38 (0.60, 3.17)	.4433	1.76 (0.76, 4.07)	.1832	
	BMI(kg/m^2^)							.0491*
	<25	3117	Reference	0.92 (0.41, 2.04)	.8354	2.28 (1.06, 4.88)	.0345*	
	≥25	7164	Reference	1.73 (0.86, 3.49)	.1231	1.42 (0.68, 2.99)	.3486	
	Calcium intake (mg)							.0749
	<815	5145	Reference	0.59 (0.25, 1.40)	.233	1.18 (0.56, 2.49)	.6575	
	≥815	5167	Reference	2.12 (1.11, 4.05)	.0236*	2.62 (1.26, 5.47)	.0101*	
	eGFR(mL/min/1.73m^2^)							.4562
	<60	668	Reference	2.79 (0.65, 11.93)	.1669	3.84 (0.90, 16.32)	.0688	
	≥60	9644	Reference	1.19 (0.67, 2.10)	.5539	1.67 (0.93, 2.99)	.0843	
Trochanter	Age (year)							.0425
	<65	8005	Reference	0.43 (0.13, 1.40)	.1599	0.63 (0.21, 1.93)	.4224	
	≥65	2307	Reference	2.58 (1.16, 5.72)	.02*	2.56 (1.13, 5.82)	.0245	
	Gender							.2931
	Male^a^	5343	Reference	1.22 (0.30, 5.02)	.7801	0.44 (0.06, 3.07)	.4083	
	Female	4969	Reference	1.70 (0.83, 3.47)	.1437	2.00 (0.97, 4.12)	.06	
	Race/ethnicity							.0425*
	Non-Hispanic White	4623	Reference	1.48 (0.53, 4.16)	.454	3.22 (1.18, 8.79)	.0225*	
	Others	5689	Reference	1.54 (0.68, 3.49)	.302	0.95 (0.39, 2.27)	.9004	
	BMI(kg/m^2^)							.9582
	<25	3117	Reference	1.53 (0.63, 3.74)	.3475	1.67 (0.67, 4.18)	.2746	
	≥25	7164	Reference	1.27 (0.51, 3.16)	.6071	1.48 (0.59, 3.74)	.4028	
	Calcium intake (mg)							.6698
	<815	5145	Reference	1.06 (0.41, 2.73)	.9038	1.21 (0.52, 2.85)	.6545	
	≥815	5167	Reference	1.90 (0.81, 4.45)	.1387	1.81 (0.68, 4.82)	.2318	
	eGFR(mL/min/1.73m^2^)							.9497
	<60	668	Reference	1.36 (0.26, 6.99)	.7153	1.89 (0.37, 9.61)	.4438	
	≥60	9644	Reference	1.49 (0.75, 2.96)	.2517	1.48 (0.72, 3.02)	.2835	
Intertrochanter	Age (year)							.1481
	<65	8005	Reference	0.35 (0.07, 1.69)	.1899	0.93 (0.23, 3.76)	.9215	
	≥65	2307	Reference	1.36 (0.60, 3.10)	.46	2.46 (1.10, 5.52)	.0285*	
	Gender							.2987
	Male^a^	5343	Reference	0.77 (0.18, 3.34)	.7263	0.46 (0.06, 3.81)	.4741	
	Female	4969	Reference	1.20 (0.52, 2.76)	.6756	2.95 (1.32, 6.60)	.0086*	
	Race/ethnicity							.1481
	Non-Hispanic White	4623	Reference	0.67 (0.25, 1.80)	.4293	2.60 (1.06, 6.39)	.0375*	
	Others	5689	Reference	1.78 (0.57, 5.58)	.3256	1.97 (0.61, 6.36)	.2552	
	BMI(kg/m^2^)							.2776
	<25	3117	Reference	0.66 (0.28, 1.57)	.3475	1.59 (0.70, 3.62)	.2726	
	≥25	7164	Reference	2.43 (0.60, 9.86)	.2157	3.66 (0.93, 14.49)	.0642	
	Calcium intake (mg)							.4335
	<815	5145	Reference	0.73 (0.21, 2.45)	.6064	1.90 (0.68, 5.28)	.2186	
	≥815	5167	Reference	1.44 (0.58, 3.57)	.4332	1.60 (0.57, 4.50)	.3744	
	eGFR(mL/min/1.73m^2^)							.9386
	<60	668	Reference	0.91 (0.17, 4.96)	.9093	2.33 (0.44, 12.23)	.3170	
	≥60	9644	Reference	1.07 (0.49, 2.37)	.86	1.94 (0.89, 4.24)	.0956	
Total femur	Age (year)							.5776
	<65	8005	Reference	0.37 (0.11, 1.32)	.1266	0.75 (0.23, 2.41)	.6266	
	≥65	2307	Reference	1.78 (0.81, 3.91)	.1506	2.86 (1.30, 6.32)	.0092*	
	Gender							.1863
	Male^a^	5343	Reference	0.52 (0.09, 3.07)	.4732	0.27 (0.02, 3.17)	.2969	
	Female	4969	Reference	1.48 (0.72, 3.05)	.2823	2.49 (1.22, 5.09)	.0123	
	Race/ethnicity							.5776
	Non-Hispanic White	4623	Reference	0.95 (0.39, 2.36)	.9167	2.24 (0.95, 5.30)	.0668	
	Others	5689	Reference	1.53 (0.57, 4.11)	.4005	2.00 (0.74, 5.44)	.1748	
	BMI(kg/m^2^)							.5712
	<25	3117	Reference	0.84 (0.37, 1.91)	.6705	1.60 (0.72, 3.57)	.2508	
	≥25	7164	Reference	1.73 (0.58, 5.16)	.323	2.48 (0.84, 7.36)	.1005	
	Calcium intake (mg)							.3791
	<815	5145	Reference	0.63 (0.22, 1.83)	.3966	1.27 (0.52, 3.11)	.6014	
	≥815	5167	Reference	1.64 (0.71, 3.75)	.2439	1.94 (0.77, 4.86)	.1591	
	eGFR(mL/min/1.73m^2^)							.8526
	<60	668	Reference	1.38 (0.27, 7.06)	.6979	2.09 (0.40, 10.88)	.3791	
	≥60	9644	Reference	1.10 (0.54, 2.25)	.7968	1.85 (0.91, 3.75)	.087	

Adjust for age, gender, race/ethnicity, smoker, BMI, eGFR, UACR, serum C-reactive protein, WBC count, NLR, serum calcium, arthritis, aspirin use, calcitonin use, biphosphonate use, DMARDs use, calcium intake and estrogen use, except for the variate for subgroup analysis.

DII: dietary inflammation index; OR: odds ratio; CI: confidence interval; BMI: body mass index; eGFR: estimated glomerular filtration rate; UACR: urine albumin-creatinine ratio; WBC: white blood cell; NLR: neutrophil to lyphocyte ratio; DMARDs: disease-modifying antirheumatic drugs.

^a^Male was not adjusted with estrogen use.

*p<.05.

## Discussion

Our results showed that higher DII, indicating diet patterns with more pro-inflammatory potential, is independently associated with BMD loss in all the femoral areas and is associated with the presence of osteoporosis in the femoral neck, the intertrochanter, and the total femur. Though this positive association was not observed in the trochanteric area in the whole population after full adjustment, subgroup analysis revealed its existence in the non-Hispanic White population, indicating that they may pay more attention on the BMD of this area than the other races. Subjects with or without kidney function impairment (eGFR < 60 mL/min/1.73 m^2^) showed no significant difference on the relationship between DII and femoral BMD or the presence of femoral osteoporosis.

Accumulating evidence has demonstrated that dietary continents could influence inflammatory cytokines secretion [12,42]. DII was developed by calculating a score for 45 food parameters reported to regulate the levels of 6 specific inflammatory biomarkers (IL-1β, IL-4, IL-6, IL-10, tumor necrosis factor-α (TNF-α), and CRP) to quantify the actual effect of diet on inflammation [17], and has been found to closely associated with several inflammatory cytokines including CRP, IL-6, and homocysteine [34,43,44]. There may be several mechanisms by which a pro-inflammatory diet leads to poor musculoskeletal health [45]. A pro-inflammatory diet may lead to enhanced oxidative stress and immune disorders which induced elevated circulating inflammatory cytokines [18]. Meanwhile, it could synergize with aging, which is also an important trigger of immune dysregulation, to induce increased production of proinflammatory cytokines that result in prolonged inflammation and adverse musculoskeletal health [45]. Elevated serum proinflammatory markers, such as TNF-α, CRP, and IL-6, are associated with various musculoskeletal conditions such as fractures [5,6], declined muscular mass and strength [46–48], and frailty [5]. Existing studies demonstrated that inflammatory cytokines, by promoting the production of receptor activators of NF-κB ligand (RANKL), can directly stimulate differentiation and overactivation of the osteoclast [49]. IL-1 and IL-6 were found to be able to interfere with bone remodeling by enhancing bone resorption and suppressing bone formation [50,51]. Another study also showed that higher CRP levels were associated with lower trabecular bone scores and bone-quality index [52].

Our result that higher DII scores were strongly related to BMD loss is consistent with many previous studies [20,23,27,53–55]. Though there is research reported that a diet with high pro-inflammatory potential was associated with an elevated risk of osteoporotic hip fracture in both men and women of an elderly Chinese population [28], most evidence has been observed that a pro-inflammatory diet pattern was significantly associated with more chance of being diagnosed as osteoporosis [27,56] and fractures [26] in women, but not in men. These data supported our finding that DII or osteoporosis diagnoses had a positive association in the femoral neck, the intertrochanter, and the total femur of the general population and, intriguingly, in the trochanteric area of the non-Hispanic White population. Chronic kidney disease (CKD) was revealed as an individual risk factor of osteoporosis, patients with CKD often suffer chronic inflammation and bone metabolism disorder [57]. Studies have revealed that CKD could negatively affect bone strength, and impaired eGFR was closely associated with an increased risk of hip fracture [58] and tibial microstructural impairment [59]. Consistently, our results have observed that the declined eGFR was related to a decreased BMD and increased chance of the presence of osteoporosis in femoral areas. However, no significant influence on the association between DII and the presence of osteoporosis by the declined eGFR was found.

As a chronic disease whose pathology is closely related to systemic inflammation, osteoporosis may be affected by various nutrition intakes [4–6]; meanwhile, the loss of bone density becomes more and more severe with time pass by. So, it is an important strategy for osteoporosis prevention to have a properly controlled overall intake of various nutrition after middle age [60]. Given the rising incidence of fractures worldwide [61], more efforts should be made to construct and implement a dietary pattern with better overall nutritional quality and anti-inflammatory properties to improve BMD loss and prevent osteoporosis.

There are several limitations that should be discussed. First, only correlation conclusions but no causal conclusions could be drawn since it is a cross-sectional study, more prospective studies are needed for further investigation. Second, most of the data for subjects from NHANES were just collected at one time point which keeps us from determining the association over time and may also induce bias in the classification of the specific population such as subjects with or without impairment of kidney function. Third, the dietary information came from the self-reported questionnaires, which might result in a recall or misclassification bias. Fourth, data from other bone areas were not collected for BMD analysis, and only the data of bone density but not femoral fractures were collected as the outcome for bone health. Fifth, the findings may not generalize to individuals under 18 years and pregnant females. Sixth, residual confounding is always a concern even after multivariable adjustment. Despite these limitations, our study observed the associations between DII and femoral bone density and bring further understanding of osteoporosis prevention.

## Conclusion

In this study, we used a large multiracial cohort from NHANES to explore the association between the DII and the BMD or the presence of osteoporosis in the femoral areas, we found a higher DII, which indicates diet patterns with more pro-inflammatory potential, is independently associated with declined femoral BMD and with the presence of trochanteric osteoporosis in the non-Hispanic White population. However, no significant difference in the relationship between DII and the presence of femoral osteoporosis was observed between subjects with or without kidney function impairment (eGFR < 60 mL/min/1.73 m^2^).

## Supplementary Material

Supplemental Material

Supplemental Material

Supplemental Material

Supplemental Material

## Data Availability

All data used in the study are publicly available online. (https://wwwn.cdc.gov/nchs/nhanes/Default.aspx and https://www.cdc.gov/nchs/data-linkage/mortality-public.htm).
