# Supplementary figures and images for: The association between dietary inflammation index and bone mineral density: results from the United States National Health and nutrition examination surveys
Source: Ren Fail. 2023 May 8;45(1):2209200. doi: 10.1080/0886022X.2023.2209200 (PMC10167883; doi:10.1080/0886022X.2023.2209200)

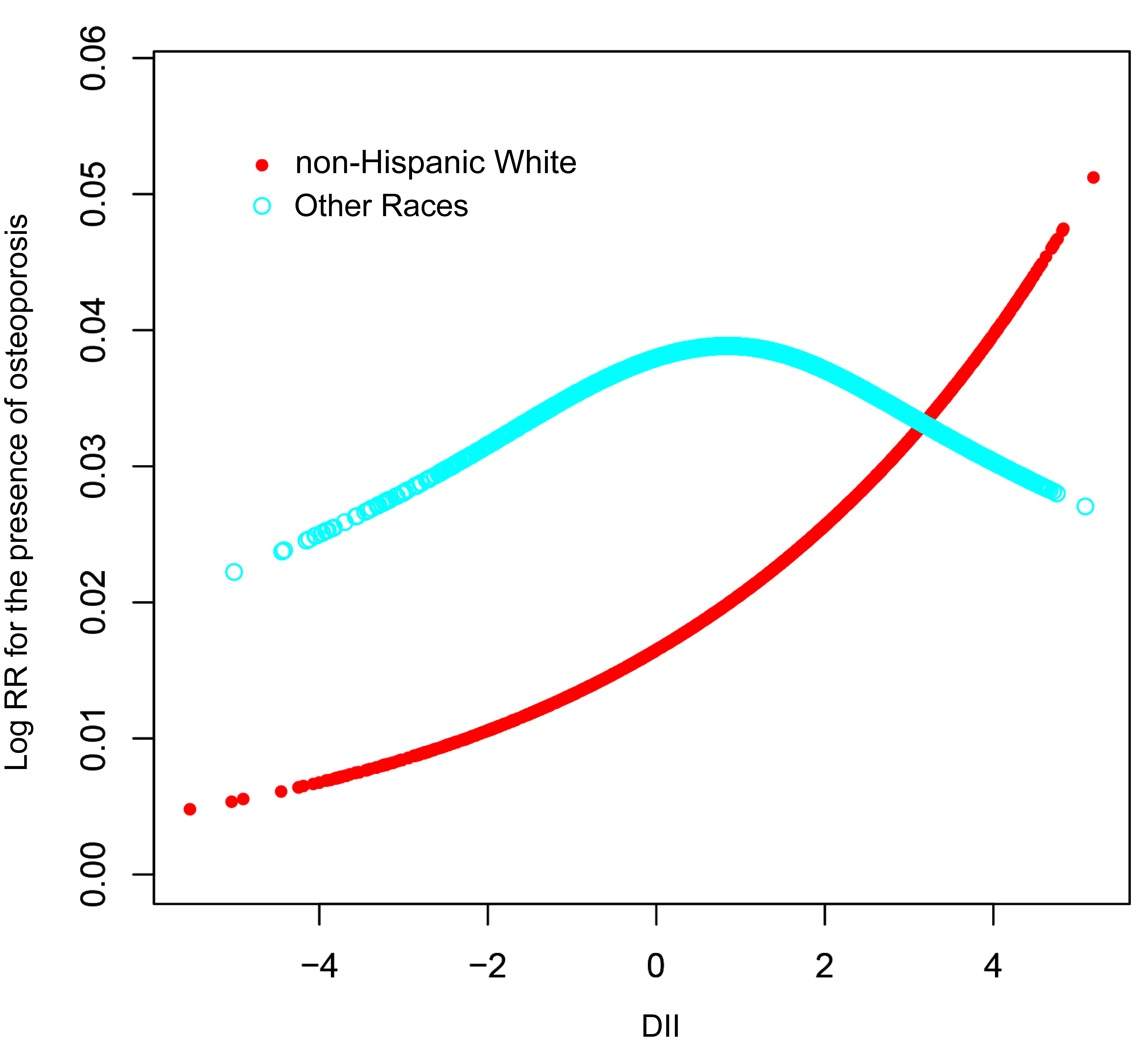

Supplement: Supplemental Material [file IRNF_A_2209200_SM6191.tif]

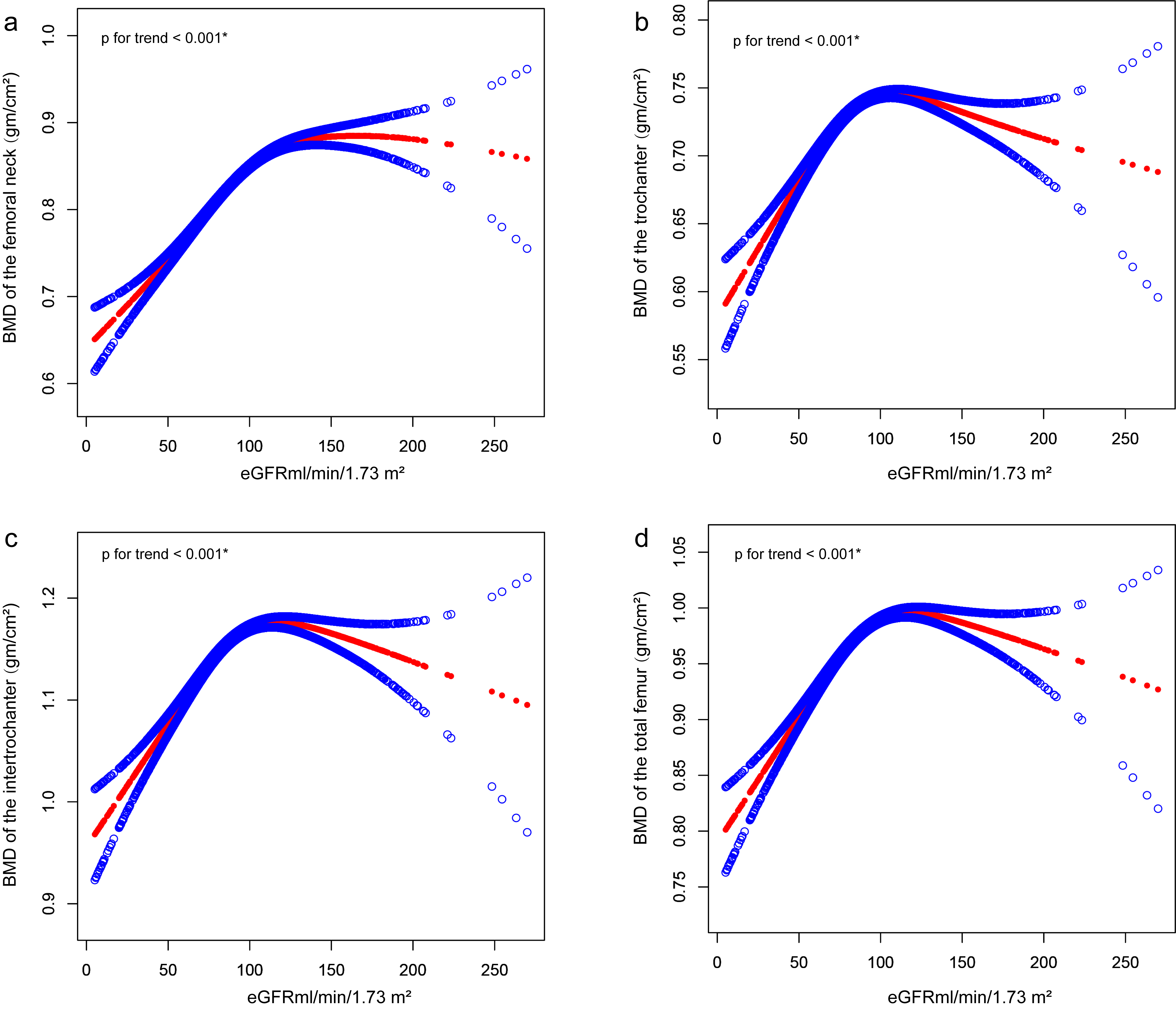

Supplement: Supplemental Material [file IRNF_A_2209200_SM6186.tif]
