# Supplementary material for: The association between dietary inflammation index and bone mineral density: results from the United States National Health and nutrition examination surveys
Source: Ren Fail. 2023 May 8;45(1):2209200. doi: 10.1080/0886022X.2023.2209200 (PMC10167883; doi:10.1080/0886022X.2023.2209200)
Supplement: Supplemental Material [file IRNF_A_2209200_SM6180.pdf]

Region of interest	eGFR (ml/min/1.73 m²)	Dietary Inflammatory Index (DII) Tertile1 <0.380	Tertile2 0.380-2.286 OR (95% CI)	Tertile2 0.380-2.286 P value	Tertile3 >2.286 OR (95% CI)	Tertile3 >2.286 P value	p for interaction
Femoral neck	<60	Reference	2.29 (1.03, 5.06)	0.0416*	3.01 (1.43, 6.35)	0.0037*	<0.001*
	>=60	Reference	1.03 (0.74, 1.43)	0.8723	1.46 (1.08, 1.99)	0.0154*	
Trochanter	<60	Reference	2.76 (0.75, 10.18)	0.1285	4.11 (1.21, 13.99)	0.0238*	<0.001*
	>=60	Reference	1.67 (1.04, 2.68)	0.0327*	2.18 (1.38, 3.43)	0.0008*	
Intertrochanter	<60	Reference	1.62 (0.40, 6.58)	0.4998	3.48 (1.01, 12.01)	0.0480*	<0.001*
	>=60	Reference	1.35 (0.80, 2.30)	0.264	2.28 (1.40, 3.70)	0.0009*	
Total femur	<60	Reference	1.84 (0.56, 6.08)	0.3182	3.06 (1.03, 9.08)	0.0435*	<0.001*
	>=60	Reference	1.11 (0.69, 1.78)	0.6777	2.01 (1.31, 3.07)	0.0013*	

Supplemental table 2. Association between eGFR and the presence of osteoporosis.

DII, dietary inflammation index; OR, odds ratio; CI, confidence interval; eGFR, estimated glomerular filtration rate.

*p < 0.05

Information Classification: General
