# Supplementary material for: The association between dietary inflammation index and bone mineral density: results from the United States National Health and nutrition examination surveys
Source: Ren Fail. 2023 May 8;45(1):2209200. doi: 10.1080/0886022X.2023.2209200 (PMC10167883; doi:10.1080/0886022X.2023.2209200)
Supplement: Supplemental Material [file IRNF_A_2209200_SM6165.pdf]

Region of interest	Subgroup	Sample size	Dietary Inflammatory Index (DII) without Vitamin D* Tertile1 <0.350	Tertile2 0.350-2.254		Tertile3 >2.286		p for interaction
				OR (95% CI)	P value	OR (95% CI)	P value	
Femoral neck	Vitamin D intake							0.338
	<3.2mcg/d	5067	1	0.73 (0.27, 2.00)	0.5461	1.49 (0.61, 3.64)	0.3857	
	≥3.2mcg/d	5245	1	1.69 (0.92, 3.12)	0.0907	2.06 (1.04, 4.08)	0.0376*	
Trochanter	Vitamin D intake							0.9315
	<3.2mcg/d	5067	1	1.14 (0.34, 3.86)	0.8338	1.40 (0.44, 4.44)	0.563	
	≥3.2mcg/d	5245	1	1.56 (0.74, 3.31)	0.2466	1.57 (0.66, 3.72)	0.3072	
Intertrochanter	Vitamin D intake							0.696
	<3.2mcg/d	5067	1	0.82 (0.17, 3.84)	0.7965	2.30 (0.58, 9.13)	0.2375	
	≥3.2mcg/d	5245	1	1.04 (0.46, 2.37)	0.918	1.65 (0.66, 4.12)	0.2861	
Total femur	Vitamin D intake							0.8132
	<3.2mcg/d	5067	1	0.79 (0.23, 2.70)	0.7078	1.34 (0.44, 4.12)	0.6092	
	≥3.2mcg/d	5245	1	1.15 (0.53, 2.49)	0.7213	1.95 (0.84, 4.51)	0.1202	

Supplemental Table 1 Subgroup analysis of the association between DII without Vitamin D intake and possibility of the presence of osteoporosis by Vitamin D intake

*This DII was calculated with energy, carbohydrate; protein; total fat; dietary fiber; cholesterol; saturated, monounsaturated, and polyunsaturated fatty acids; ω-3 and ω-6 polyunsaturated fatty acids; vitamin A, B1, B2, B3, B6, B12, C, and E; folic acid; alcohol; β-carotene; caffeine; iron; magnesium; zinc; and selenium, but not Vitamin D intake.

Adjust for age, gender, race/ethnicity, smoker, BMI, eGFR, UACR, serum C-reactive protein, WBC count, NLR, serum calcium, arthritis, aspirin use, calcitonin use, diphosphonate use, DMARDs use, calcium intake and estrogen use, except for Vitamin D intake.

DII, dietary inflammation index; OR, odds ratio; CI, confidence interval; BMI, body mass index; eGFR, estimated glomerular filtration rate; UACR, urine albumin-creatinine ratio; WBC, white blood cell; NLR, neutrophil to lyphocyte ratio; DMARDs, disease-modifying antirheumatic drugs.

*p < 0.05

Information Classification: General
